# Pan-cancer analysis and *in vitro* validation of the oncogenic and prognostic roles of *AURKA* in human cancers

**DOI:** 10.3389/fonc.2023.1186101

**Published:** 2023-10-27

**Authors:** Chuang Yang, Patrick Sven Plum, Ines Gockel, René Thieme

**Affiliations:** Department of Visceral, Transplant, Thoracic and Vascular Surgery, University Hospital of Leipzig, Leipzig, Germany

**Keywords:** AURKA, pan-cancer, esophageal adenocarcinoma (EAC), biomarker, spheroids

## Abstract

**Background:**

Aurora kinase A (AURKA) plays a pivotal role in regulating cell mitosis and tumor progression. However, its prognostic significance across diverse cancer types remains relatively unexplored.

**Methods:**

We conducted a comprehensive analysis of AURKA expression in various cancers using data from The Cancer Genome Atlas, Genotype-Tissue Expression, and The Human Protein Atlas databases. Our investigation encompassed an exploration of the associations between AURKA expression and clinical characteristics, shedding light on potential functional roles of AURKA. Additionally, we delved into the relationship between AURKA and the tumor microenvironment. To substantiate the role of AURKA, we carried out *in vitro* experiments in esophageal adenocarcinoma (EAC), prostate cancer (PRAD), and pancreatic cancer (PAAD) cells.

**Results:**

Our analysis revealed that AURKA is prominently overexpressed in a majority of the cancer types under investigation. Elevated AURKA expression correlated closely with poorer prognosis and advanced tumor stages. AURKA was found to be associated with key pathways involved in the cell cycle and arachidonic acid metabolism. Moreover, AURKA expression exhibited significant correlations with immunoregulatory genes and immune cell profiles. Notably, *in vitro* experiments demonstrated that silencing AURKA expression resulted in reduced cell viability in EAC, PRAD, and PAAD cells, as well as a decrease in clone formation, cell cycle elongation, diminished cell invasion and reduced spheroid size in EAC cells (OE33 and OE19).

**Conclusion:**

Our study elucidates the oncogenic role of AURKA and underscores its prognostic value across a spectrum of cancers, including EAC. These findings suggest that AURKA holds promise as a predictive biomarker for EAC and various other tumor types.

## 1 Introduction

The high global incidence of esophageal cancer (ESCA) poses a significant threat to human life. Esophageal adenocarcinoma (EAC), a common subtype, has rapidly increased in incidence, particularly in high-income countries (1). Due to the absence of specific early symptoms, distinctive physical features, and effective diagnostic markers, patients with EAC are often diagnosed at an advanced stage, resulting in a generally poor clinical prognosis (2). Consequently, the identification of effective prognostic molecular markers becomes imperative, as it holds the potential to facilitate earlier diagnosis and novel treatment strategies, ultimately extending the overall survival (OS) of patients with EAC.

Aurora kinase A (AURKA) plays a crucial role as a serine/threonine kinase family member, influencing cytokinesis and spindle formation throughout the cell cycle and mitosis (3, 4). Aberrant expression of AURKA has been observed in various human cancers, spanning gastric (5), esophageal (6), colon (7), ovarian (8), and pancreatic (9) cancers. Furthermore, elevated AURKA expression has been linked to poor prognosis and drug resistance (10, 11).

While immunotherapy is a promising cancer treatment in clinical oncology, only a small percentage of patients benefit from it due to immune resistance exhibited by their tumor cells (12). Factors such as tumor mutational burden (TMB) and microsatellite instability (MSI) are pivotal in driving resistance to immunotherapy (13). The tumor immune microenvironment (TIME) also significantly influences tumor progression and the effectiveness of cancer therapy (14). Immune cell infiltration and function are intricately tied to the TIME. Notably, the inhibition of AURKA has been shown to reshape the TIME by depleting tumor-promoting myeloid cells and enriching anti-cancer T-lymphocytes, leading to the regression of mammary tumors in mice (15). Additionally, AURKA inhibition enhances T-cell cytotoxicity and augments anti-tumor immunity *in vitro* (16).

Several studies have indicated that AURKA plays an oncogenic role in cancer. However, the precise role and clinical relevance of AURKA in tumorigenesis remain unclear. For instance, et al. investigated AURKA’s role in 13 different tumors but primarily focused on gene expression variations and drug resistance, with limited consideration for prognosis (17). Furthermore, their study lacks experimental validation. Additionally, in ESCA, most studies have concentrated on AURKA’s role in esophageal squamous cell carcinoma, with limited attention given to EAC (6, 18, 19). Particularly, there is a paucity of literature that comprehensively synthesizes the clinical significance and molecular biological functions of AURKA in EAC and other malignancies.

In this study, we initiated our exploration by examining AURKA’s expression, prognostic significance, and associated immune characteristics using a pan-cancer approach based on The Cancer Genome Atlas (TCGA) database. We then delved into the interactions between AURKA, immune cell infiltration, and immunoregulatory factors, with validation from GEO datasets. Finally, we substantiated the biological role of AURKA in three different tumor cell lines, encompassing EAC, PRAD, and PAAD, through various cellular phenotype experiments. Our findings strongly support the utility of AURKA as a prognostic marker across various cancer types.

## 2 Materials and methods

### 2.1 Data acquisition and processing

We utilized pan-cancer RNA-sequencing data and clinical information from TCGA (20) and acquired GTEx data from the UCSC Xena website (http://xena.ucsc.edu/) (21). Information regarding TMB and MSI was extracted from the TCGA dataset. Additionally, we gathered datasets from GEO databases (https://www.ncbi.nlm.nih.gov/geo/) for validation purposes in our pan-cancer analysis, specifically GSE19750, GSE13507, GSE42568, GSE39582, GSE13898, GSE6631, GSE66858, GSE15641, GSE167573, GSE26574, GSE106291, GSE50161, GSE43378, GSE76427, GSE30219, GSE51024, GSE183088, GSE26712, GSE28735, GSE62452, GSE32571, GSE116918, GSE87211, GSE21122, GSE30930, GSE15605, GSE65904, and GSE26899, GSE84437, GSE65144, and GSE17025.

### 2.2 *AURKA* expression analysis

We obtained cancer tissue data from the TCGA dataset and normal tissue data from both TCGA and GTEx datasets. Differences in AURKA expression between cancer and normal tissues were assessed using the “rma” function in R software, with significance defined as p < 0.05. A comprehensive list of cancer types and their corresponding abbreviations used in our study are provided in **Supplementary Table 1**. Protein expression of AURKA in normal and pathological tumor tissues was analyzed using THPA (http://www.proteinatlas.org/), which provides immunohistochemistry results for AURKA protein expression in various tissues (22).

### 2.3 Correlation between survival and *AURKA* levels in pan-cancer

To evaluate the prognostic value of AURKA in patients with cancer from TCGA database, we conducted Kaplan–Meier (KM) analyses based on the median expression level of AURKA. Additionally, we performed a univariate Cox regression analysis to investigate the predictive significance of AURKA.

### 2.4 Gene set enrichment analysis

We employed the “cluster Profiler” package for Gene Set Enrichment Analysis (GSEA) to explore potential pathways associated with AURKA expression (23). Biological functions were examined based on KEGG and HALLMARK pathways.

### 2.5 Immune cell infiltration analysis

The relative numbers of immune cells were obtained from the TIMER2.0 database (http://timer.cistrome.org/) using the CIBERSORT method (24). We analyzed the association between AURKA expression and the ESTIMATE score and immune score in the tumor microenvironment (TME) using SangerBox (http://sangerbox.com/Tool) (25). Spearman’s rho test was employed to detect associations between AURKA expression and the immune score and ESTIMATE score.

### 2.6 Copy number variation and tumor immune dysfunction and exclusion

Copy number variations (CNVs) are linked to tumor progression, with higher CNV scores indicating increased aggressiveness (26). We acquired CNV data from the cBioPortal database (https://www.cbioportal.org/) (27). The TIDE score, which assesses tumor immune escape and predicts responses to immune checkpoint therapy (28), was utilized to explore the correlation between AURKA expression, T-cell lesions, and immunotherapeutic responses. We analyzed these relationships using data from the TIDE database (http://tide.dfci.harvard.edu/).

### 2.7 Immunomodulator analysis

Our analysis of the correlation between AURKA and immunomodulators, including immune-activating genes, immunosuppressive genes, chemokines, and chemokine receptor genes, was conducted using the TISIDB database (http://cis.hku.hk/TISIDB/) (29).

### 2.8 Cell lines

The esophageal squamous mucosa cells EPC1 and EPC2 were generously provided by Dr. Hiroshi Nakagawa (30). Prostate cancer cells DU145, pancreatic cancer cells PaTu8988t, and EAC cells OE33 and OE19 were purchased from Sigma-Aldrich (Taufkirchen, Germany). These cells were cultured in RPMI-1640 medium (Thermo Fisher Scientific, Germany) supplemented with 10% fetal bovine serum (Sigma-Aldrich, Germany).

### 2.9 Cell transfection

AURKA was silenced using antisense LNA GapmeRs (cat. no. 339511 LG00799137-DDA, Qiagen, Germany; sequence: 5′-T*C*T*A*G*C*T*G*T*A*A*T*A*A*G*T-3′). Following the manufacturer’s guidelines, cells were transfected with Lipofectamine™ 2000 transfection reagent and Plus™ reagent (Thermo Fisher Scientific, Germany). Initially, 25 × 10^4^ cells were seeded into six-well plates containing 2 ml of RPMI-1640 medium and cultured for 24 hours. The AURKA antisense LNA GapmeRs, as well as negative control and mock control, were designated as GapmeR, NC, and MC, respectively. Subsequently, 256 µl of optiMEM, 2.1 µl of Lipofectamine™ 2000, and 2.1 µl of Plus™ reagent were added to each tube, followed by the addition of 10.5 µl of the working solution (2 µM), with incubation at room temperature for 30 minutes. Finally, 1742 µl of complete medium and 258 µl of mixed transfection reagents were added to each well and cultured for 24 or 48 hours.

### 2.10 cDNA-synthesis and qRT-PCR

Total RNA was extracted using the RNeasy Plus Mini Kit (Qiagen, Germany). The reverse transcription reaction was carried out using the RevertAid RT Kit (Thermo Fisher Scientific, Germany). AURKA expression was quantified through qRT-PCR, with β-actin serving as the normalization reference (SYBR Green JumpStart Taq ReadyMix Kit, Sigma-Aldrich, Germany; AURKA: forward primer, GTCCCTGAGTGTCCTTGGC, reverse primer, GCAATGGAGTGAGACCCTCT; β-actin: forward primer, GTCTTCCCCTCCATCGTG, reverse primer, AGGGTGAGGATGCCTCTCTT).

### 2.11 Western blot

For protein extraction, RIPA buffer was used, and the protein concentration was assessed following Bradford’s method. Total protein (20-µg sample) was separated using 12% sodium dodecyl sulfate polyacrylamide gels and transferred onto nitrocellulose membranes. Subsequently, the membranes were blocked with 5% milk powder and incubated with specific primary antibodies at 4°C overnight (AURKA, 1:1000 dilution, #14475; Cell Signaling Technology, USA; β-actin, 1:1000 dilution; A1978 Sigma-Aldrich, Germany), followed by incubation with a conjugated secondary antibody. Protein bands were visualized using an ECL chemiluminescence detection reagent (Millipore, Billerica, MA, USA) and analyzed and quantified using ImageJ software.

### 2.12 Cell viability assay

A total of 5,000 cells were seeded in 96-well plates and incubated for 24 hours. Subsequently, cells were treated with Aur-I (Aurora-A Inhibitor I), a specific inhibitor for AURKA (Sigma-Aldrich, Germany) or AURKA GapmeR, and incubated for 24 or 48 hours, respectively. Cells were stained with PrestoBlue™ cell viability reagent (Thermo Fisher Scientific, Germany) at 37°C for 10 minutes, and cell viability was assessed using SpectraMax M5 (Molecular Devices, Sunnyvale, CA, USA).

### 2.13 Colony formation assay

In a six-well plate, 500 cells were seeded and incubated for 24 hours. The medium was subsequently replaced with Ctr (0.1% dimethyl sulfoxide [DMSO]), 1 or 5 µM Aur-I, or AURKA GapmeR, and cells were incubated for 24 hours. The medium was then removed, and fresh medium was added for an additional 10-12 days. Cells were fixed with 3.7% paraformaldehyde, stained with 0.5% crystal violet, and the number of colonies was quantified using ImageJ software.

### 2.14 Cell cycle assay

Cells were seeded and treated with Aur-I (1 µM and 5 µM) or AURKA GapmeR for 24 hours, followed by harvesting. Fixation was performed using 70% ethanol overnight. The cells were subsequently washed with PBS, stained with a propidium iodide staining solution, and analyzed through flow cytometry (BD LSRFortessa™ Cell Analyzer, BD Biosciences, Germany).

### 2.15 Boyden chamber assay

The Boyden chamber assay was carried out using 24-well trans-well inserts (ThinCert™, Greiner Bio-One, Germany). Cells were treated with AURKA GapmeR for 48 hours. The lower surface of the insert was coated with 10 µg/ml fibronectin and incubated at 37°C for 2 hours. Cells were seeded (approximately 5,000 cells) into the upper chamber in serum-free RPMI medium, while 500 µl of serum-containing medium was transferred to the lower chamber. Subsequently, cells were fixed with 3.7% paraformaldehyde, stained with 0.1 µg/ml DAPI (4′,6-diamidino-2-phenylindole) for 24 hours, and imaged using fluorescence microscopy (Axio-Observer, Zeiss, Germany). Cells from randomized regions of three independent experiments were counted. Cell counts were determined using ImageJ software.

### 2.16 3D spheroids cultures

Briefly, 3,500 cells (OE33 and OE19) were seeded into 96-well plates (X40 Mikrotiterplates (inertGrade), Brandt, Germany) and incubated for 24 hours. Cells were then treated with Aur-I (1 µM, 5 µM). Spheroids were monitored and measured every 4 hours using CELLCYTE X™ (CYTENA, Germany).

### 2.17 Statistical analysis

Bioinformatics analyses were conducted using R software (version 3.6.1). Group differences were assessed using unpaired Student’s t-test or one-way analysis of variance (ANOVA). Correlation between AURKA and variables, including immune score, ESTIMATE score, TMB, MSI, and TIDE, was evaluated using Spearman or Pearson correlation tests. Visualizations for *in vitro* experiments were generated using GraphPad Prism (version 9.0; La, Jolla, CA, USA). For differences obtained, the data are expressed as mean ± standard error of the mean. Differences were considered statistically significant at p-value < 0.05.

## 3 Results

### 3.1 *AURKA* is aberrantly expressed in multiple cancer types

**Figure 1:**
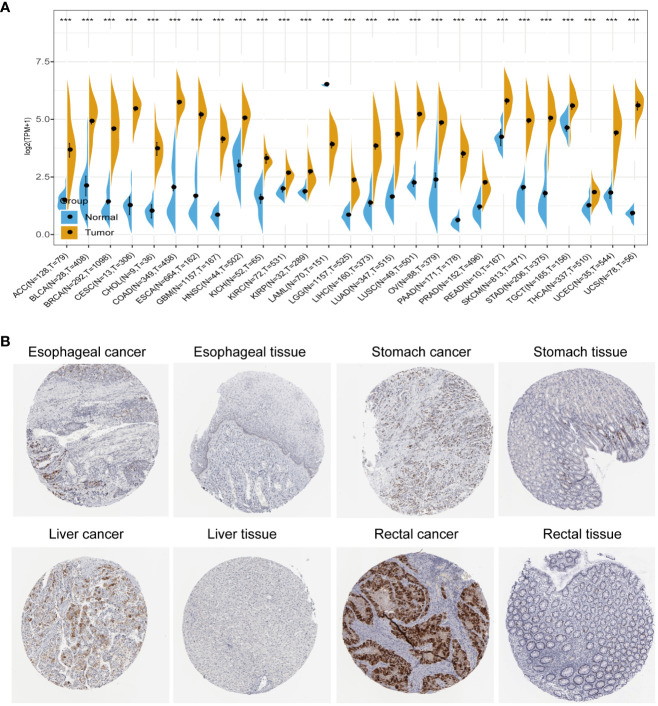
Pan-cancer analysis of AURKA expression. **(A)** Differential mRNA expression of AURKA in cancer tissues compared with normal tissues from TCGA and GTEx databases. **(B)** Immunohistochemical staining of AURKA in tumor and normal tissues. ***p < 0.001.

We conducted a comprehensive analysis by combining normal samples from the GTEx dataset with tumor samples from the TCGA dataset to investigate the differential expression of AURKA across 27 different human cancer types. Our analysis revealed that AURKA exhibited high expression in 26 cancer types, including ACC, BLCA, and others (all p<0.001), as depicted in **Figure 1A**. Notably, AURKA expression was notably low in LAML tumor tissues (p<0.001). Additionally, immunohistochemistry results sourced from the THPA database supported our findings, showing elevated protein levels of AURKA in esophageal, stomach, liver, and rectal cancer tissues (**Figure 1B**).

### 3.2 Prognostic value of *AURKA* in multiple human cancer types

To establish the prognostic significance of AURKA, we employed KM and univariate Cox regression analyses using the TCGA pan-cancer data. Our analysis of OS revealed that higher AURKA expression was associated with worse prognoses in most cancers, including ACC, ESCA, LGG, PCPG, THCA, BLCA, LIHC, PRAD, THYM, BRCA, HNSC, LUAD, UCEC, KICH, LUSC, SARC, KIRC, MESO, SKCM, UVM, KIRP, and PAAD (all p<0.05), as shown in **Supplementary Figure 1A**. Intriguingly, AURKA appeared to function as a protective factor in READ (p=0.025), COAD (p=0.019), OV (p=0.048), STAD (p=0.027), and LAML (p=0.027). Furthermore, our analysis of disease-specific survival (DSS) demonstrated that AURKA was a risk factor in ACC, ESCA, LGG, PCPG, THCA, BLCA, LIHC, PRAD, BRCA, HNSC, LUAD, UCEC, KICH, LUSC, SARC, KIRC, MESO, SKCM, UVM, KIRP, and PAAD (all p<0.05), while high AURKA expression correlated negatively with prognoses in COAD (p=0.027), OV (p=0.02), and STAD (p=0.037; **Supplementary Figure 1B**).

**Figure 2:**
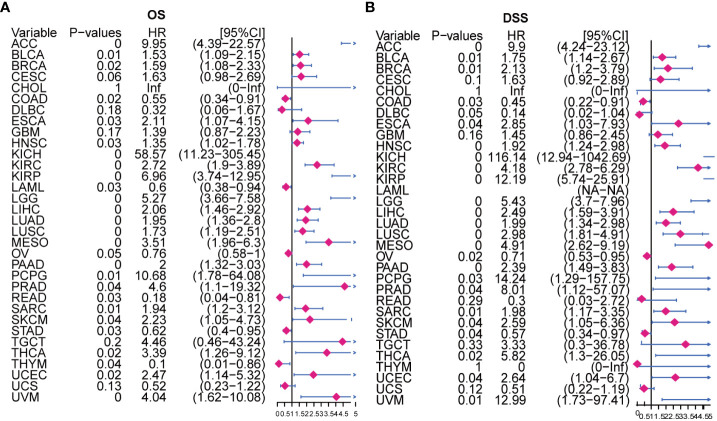
Univariate Cox regression analysis of AURKA expression. **(A)** The Forest map illustrates the correlation of AURKA expression with overall survival (OS) in various cancers. **(B)** The Forest map shows the correlation of AURKA expression with disease-specific survival (DSS) in multiple cancers. OS, overall survival. DSS, disease-specific survival.

**Figure 3:**
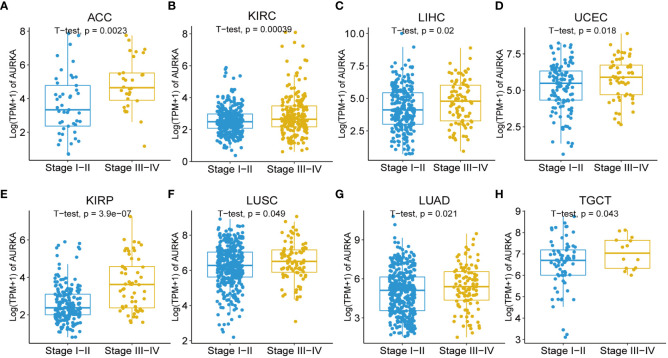
Association between AURKA expression and tumor stages. **(A–H)** AURKA expression in different stages of ACC, KIRC, LIHC, UCEC, KIRP, LUSC, LUAD, and TGCT. ACC, adrenocortical carcinoma. KIRC, kidney renal clear cell carcinoma. LIHC, liver hepatocellular carcinoma. UCEC, uterine corpus endometrial carcinoma. KIRP, kidney renal papillary cell carcinoma. LUSC, lung squamous cell carcinoma. LUAD, lung adenocarcinoma. TGCT, testicular germ cell tumors.

Moreover, our univariate Cox regression analysis for OS identified AURKA expression as an independent risk factor for OS in ACC, BLCA, BRCA, COAD, ESCA, HNSC, KICH, KIRC, KIRP, LAML, LGG, LIHC, LUAD, LUSC, MESO, PAAD, PCPG, PRAD, READ, SARC, SKCM, STAD, THCA, THYM, UCEC, and UVM (**Figure 2A**). In terms of DSS, AURKA expression was associated with 24 tumor types, with higher AURKA expression acting as a protective factor in COAD (p=0.003), OV (p=0.02), and STAD (p=0.04; **Figure 2B**). We further explored the relationship between AURKA and the UICC tumor stage, revealing that AURKA was linked to advanced tumor stages in ACC (p=0.0023), KIRC (p<0.001), LIHC (p=0.02), UCEC (p=0.018), KIRP (p<0.001), LUSC (p=0.049), LUAD (p=0.021), and TGCT (p=0.043; **Figure 3**). These findings strongly suggest that AURKA is closely associated with patient outcomes and holds potential as a prognostic factor across a wide range of human cancer types.

### 3.3 GSEA of *AURKA* expression

**Figure 4:**
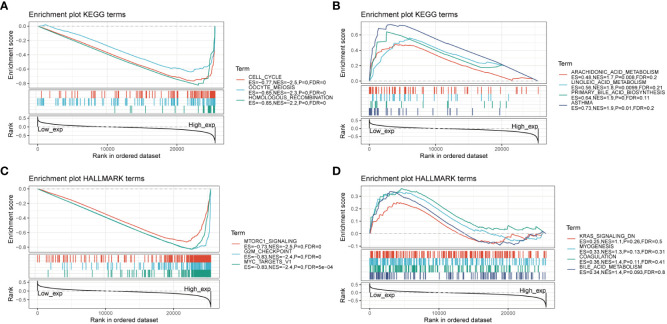
GSEA of AURKA linked with KEGG and HALLMARK pathways. **(A, B)** GSEA results of AURKA for its negative **(A)** and positive **(B)** associations with KEGG pathways. **(C, D)** GSEA results of AURKA for its negative **(C)** and positive **(D)** associations with HALLMARK pathways.

To gain insights into the biological pathways involving AURKA, we conducted GSEA focusing on KEGG and HALLMARK pathways (**Figure 4**). Our KEGG analysis unveiled a negative correlation between AURKA expression and pathways related to the cell cycle, oocyte meiosis, and homologous recombination (**Figure 4A**). Conversely, pathways such as arachidonic acid metabolism, linoleic acid metabolism, and asthma displayed a positive association with AURKA expression (**Figure 4B**). Similarly, the HALLMARK pathway analysis demonstrated that AURKA was negatively linked to pathways such as mTORC1 signaling, G2M checkpoint, and MYC target genes (**Figure 4C**), while it exhibited a positive correlation with pathways such as KARS signaling DN, myogenesis, coagulation, and bile acid metabolism (**Figure 4D**). These results underscore AURKA’s role in regulating the cell cycle and bile acid metabolism.

### 3.4 Association between *AURKA* and immune infiltration analysis

To gain a deeper understanding of the relationship between AURKA and tumor immunity, we investigated the association between AURKA expression and both ESTIMATE and immune scores in various human cancers. Our analysis revealed significant correlations between AURKA expression and the ESTIMATE score in a range of cancers, including ACC, BRCA, CESC, COAD, ESCA, GBM, HNSC, KIRC, KIRP, LGG, LIHC, LUAD, LUSC, PAAD, READ, SARC, SKCM, STAD, TGCT, THCA, and UCEC (**Supplementary Figure 2**). Additionally, AURKA exhibited correlations with the immune score in ACC, CESC, COAD, ESCA, GBM, KICH, KIRC, KIRP, LUAD, LUSC, PAAD, READ, SARC, SKCM, STAD, TGCT, THCA, and UCEC (**Supplementary Figure 3**).

**Figure 5:**
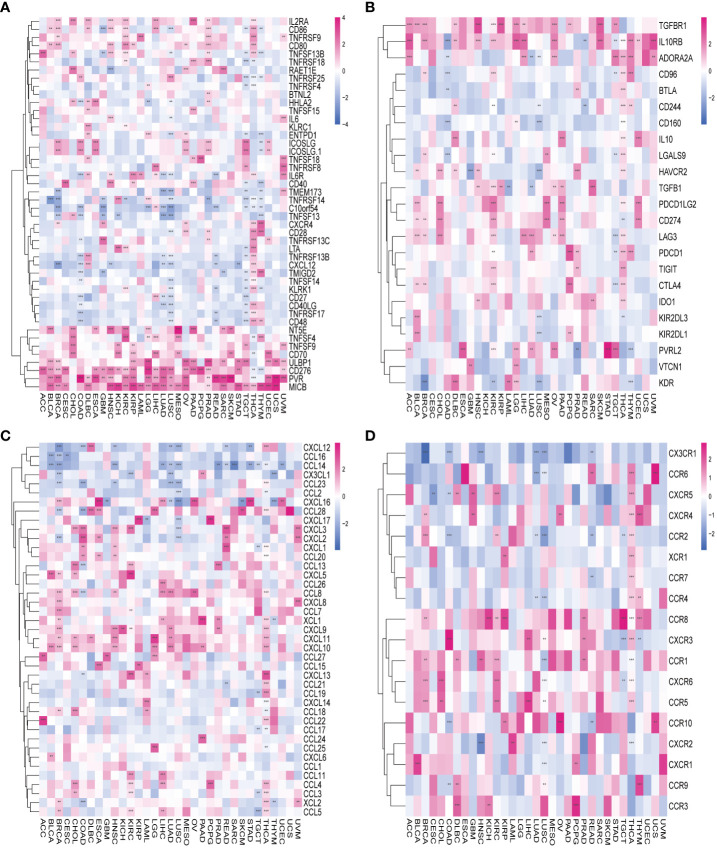
Association between AURKA and immunoregulation-related genes in human cancers. **(A)** Correlation between AURKA and immune-activating genes. **(B)** Correlation between AURKA and immunosuppressive genes. **(C)** Correlation between AURKA and chemokine-related genes. **(D)** Correlation between AURKA and chemokine receptor-related genes. **p< 0.01; ***p < 0.001.

To delve deeper into the association between AURKA and TIME), we explored the correlation between AURKA expression and immunomodulators (**Figure 5**). In terms of immune-activated genes, AURKA exhibited positive correlations with MICB, PVR, CD276, and ULBP1 and negative correlations with C10orf54 in most cancers (**Figure 5A**). Concerning immune inhibitors, AURKA displayed a positive correlation with IL10RB in most cancers but a negative correlation in COAD (**Figure 5B**). We further investigated the relationship between AURKA and chemokines and their relevant receptors, revealing a significant negative correlation between AURKA and CCL14 in most cancers (**Figure 5C**). Notably, in THCA, we observed a positive correlation between AURKA and multiple chemokine receptor genes (**Figure 5D**). Additionally, our analysis using the TIMER2 database indicated a positive association between AURKA expression and the levels of activated CD4+ T-cells and eosinophil cells in tumors, while a negative correlation was observed with the level of activated mast cells in tumors (**Supplementary Figure 4**).

### 3.5 Correlation of TMB, MSI, TIDE, and CNV with *AURKA* expression

**Figure 6:**
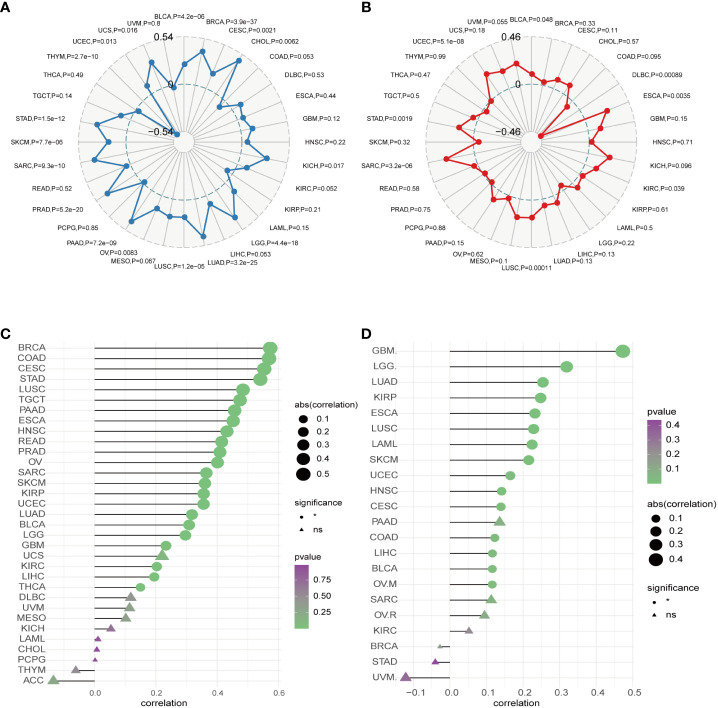
Correlation between AURKA expression and TMB, MSI, CNV, and TIDE. **(A)** Relationship between AURKA expression and tumor mutation burden (TMB) in various cancer types. **(B)** Relationship between AURKA expression and microsatellite instability (MSI) in various cancer types. **(C)** Relationship between AURKA expression and copy number variation (CNV) in various cancer types. **(D)** Relationship between AURKA expression and tumor immune dysfunction and exclusion (TIDE) in various cancer types. TMB, tumor mutation burden. MSI, microsatellite instability. CNV, copy number variation. TIDE, tumor immune dysfunction, and exclusion.

TMB and MSI are pivotal factors in tumorigenesis and progression and serve as essential biomarkers for immunotherapy. Our analysis unveiled a positive correlation between AURKA expression and TMB in several human cancers, including UCS, BRCA, CHOL, KICH, LGG, LUAD, PAAD, PRAD, SARC, and STAD (all p<0.05). Conversely, AURKA expression exhibited a negative correlation with TMB in THYM (**Figure 6A**). In terms of MSI, we observed a significant correlation between AURKA expression and the MSI score in specific cancers such as UCEC, ESCA, and SARC, while a low AURKA expression inversely correlated with MSI in DLBC (p<0.001; **Figure 6B**).

Furthermore, we explored the connection between AURKA expression and copy number variations (CNV). Our analysis demonstrated a robust correlation between AURKA expression and CNV in most cancers (**Figure 6C**), highlighting the widespread presence of AURKA and relevant CNV alterations in tumors. Finally, we employed the TIDE score to assess the potential efficacy of immune checkpoint inhibitors (ICIs) in cancer treatment. Our study revealed a strong correlation between AURKA expression and the TIDE score in cancers such as GBM, LGG, LUAD, KIRP, ESCA, LUSC, LAML, SKCM, UCEC, HNSC, CESC, COAD, LIHC, BLCA, and OVM (**Figure 6D**). These findings suggest that AURKA expression could serve as a reference marker for predicting the response to ICI therapy in cancer patients.

### 3.6 Validation of AURKA expression and prognosis in GEO cohorts

**Figure 7:**
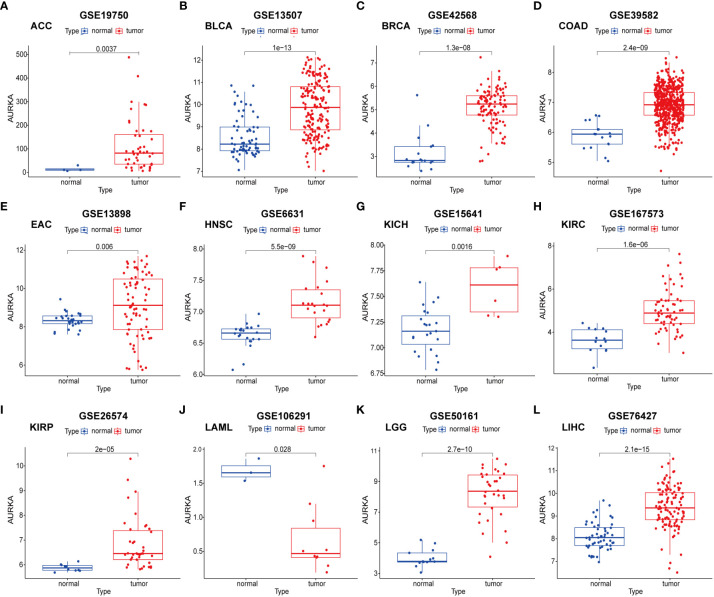
External validation of AURKA expression in GEO cohorts. **(A–L)** ACC, BLCA, BRCA, COAD, EAC, HNSC, KICH, KIRC, KIRP, LAML, LGG, and LIHC.

**Figure 8:**
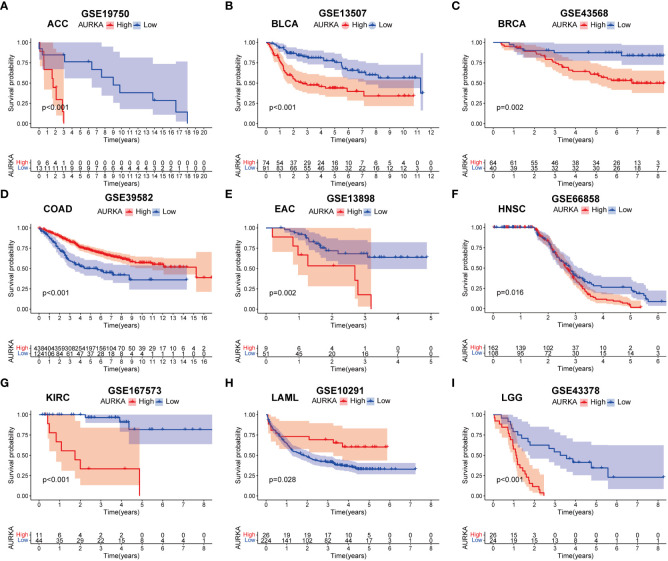
External validation of AURKA prognosis in GEO cohorts. **(A–I)** ACC, BLCA, BRCA, COAD, EAC, HNSC, KIRC, LAML, and LGG.

To validate the robustness of AURKA expression and its prognostic value, we analyzed pan-cancer data from GEO cohorts. As depicted in **Figure 7** and **Supplementary Figure 5**, AURKA exhibited higher expression in tumor tissues than in normal tissues across various tumors, including ACC, BLCA, BRCA, COAD, EAC, HNSC, KICH, KIRC, KIRP, LGG, LIHC, LUAD, MESO, OV, PAAD, PRAD, READ, SARC, SKCM, STAD, THCA, and UCEC, with the exception of LAML. Moreover, high AURKA expression was consistently associated with worse outcomes in ACC (**Figure 8A**), BLCA (**Figure 8B**), BRCA (**Figure 8C**), EAC (**Figure 8E**), HNSC (**Figure 8F**), KIRC (**Figure 8G**), LGG (**Figure 8I**), LUAD (**Supplementary Figure 6A**), MESO (**Supplementary Figure 6B**), PAAD (**Supplementary Figure 6D**), PRAD (**Supplementary Figure 6E**), SARC (**Supplementary Figure 6G**), and SKCM (**Supplementary Figure 6H**). In contrast, high AURKA expression correlated positively with the prognosis in COAD (**Figure 8D**), LAML (**Figure 8H**), OV (**Supplementary Figure 6C**), READ (**Supplementary Figure 6F**), and STAD (**Supplementary Figure 6I**). These findings corroborate our analysis of TCGA data.

We observed AURKA overexpression in various cancers, including ESCA (**Figure 1**), and its close association with patients’ OS and disease-free survival (DFS; **Supplementary Figures 1A, B**). AURKA also exhibited significant correlations with ESTIMATE and immune scores, TMB, MSI, CNV, and the TIDE score in most cancers, including ESCA (**Supplementary Figures 2**, **3**; **Figure 6**). These findings suggest that AURKA may play a pivotal role in the immune response within tumor progression and in predicting the clinical outcomes of ESCA and other cancers. Moreover, higher AURKA expression was more frequently observed in patients under 65 years of age and in those at the M1 phase in EAC (**Supplementary Figure 7**). We subsequently shifted our focus to validate and explore the function of AURKA in ESCA, particularly in EAC cells.

### 3.7 Functional validation of *AURKA in vitro*

**Figure 9:**
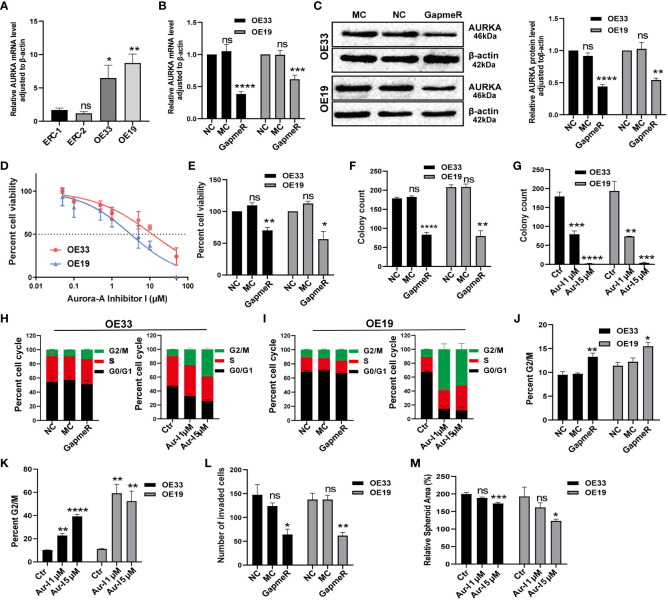
Validation of AURKA function in EAC cell lines. **(A)** AURKA expression in esophageal normal mucosa cell lines compared to OE33 and OE19, n = 6 biological replicates. **(B)** AURKA mRNA level in OE33 and OE19 cells transfected with NC, MC, and AURKA-GapmeR, n = 6 biological replicates. **(C)** AURKA protein levels in OE33 and OE19 cells after transfection with AURKA-GapmeR, n = 3 biological replicates. **(D)** Cell viability of OE33 and OE19 cells after treatment with Aur-I, n = 3 biological replicates. **(E)** Cell proliferation of OE33 and OE19 cells after transfection, n = 3 biological replicates. **(F)** Colony formation ability of OE33 and OE19 cells after transfection, n = 3 biological replicates. **(G)** Colony formation ability of OE33 and OE19 cells after treatment with Aur-I (1 µM and 5 µM), n = 3 biological replicates. **(H)** Cell cycle of OE33 cells after transfection and treatment with Aur-I, n = 3 biological replicates. **(I)** Cell cycle of OE19 cells after transfection and treatment with Aur-I (1 µM and 5 µM), along with quantified results of the G2/M phase in OE33 and OE19 after treatment with NC, MC, and AURKA-GapmeR **(J)** and Aur-I **(K)**, n = 3 biological replicates. **(L)** Boyden chamber assay of OE33 and OE19 cells after cell transfection, n = 3 biological replicates. **(M)** OE33 and OE19 spheroids treated with vehicle control (0.1% DMSO) or Aur-I (1 µM and 5 µM), n = 6 biological replicates. Data are presented as mean ± standard error of the mean. *p <0.05, **p < 0.01; ***p <0.001; ****p < 0.0001; ns, not significant. Aur-I, Aurora-A Inhibitor I.

To begin, we compared AURKA expression between normal esophageal mucosa and EAC cell lines. Quantitative real-time polymerase chain reaction (qRT-PCR) results demonstrated that AURKA was highly expressed in OE33 and OE19 cells derived from EAC when compared to squamous epithelium cell lines (**Figure 9A**). Knockdown of AURKA using AURKA GapmeR in OE33 and OE19 cells led to decreased expression of both AURKA mRNA and protein levels (**Figures 9B, C**). Subsequently, a cell viability assay revealed that inhibiting AURKA using Aur-I or AURKA GapmeR resulted in suppressed cell viability (**Figures 9D, E**). We also investigated the role of AURKA in prostate cancer (DU145) and pancreatic cancer (PaTu8988t) cells. Treating these cells with AURKA-GapmeR or Aur-I resulted in decreased AURKA mRNA expression and reduced cell viability in both prostate cancer (**Supplementary Figures 8A–C**) and pancreatic cancer cells (**Supplementary Figures 8D–F**). Further molecular and functional analyses were conducted in EAC cell lines OE33 and OE19. The colony formation assay demonstrated a significant reduction in the number of cell colonies following AURKA inhibition (**Figures 9F, G**, and **Supplementary Figure 9A**). To explore the impact of AURKA inhibition on the cell cycle, OE33 and OE19 cells were treated with AURKA-GapmeR or Aur-I for 24 hours. These results indicated that AURKA inhibition arrested the growth of OE33 and OE19 cells within the G2/M phase (**Figures 9H–K**, and **Supplementary Figure 9B**). Furthermore, the Boyden chamber assay revealed a decrease in cell invasiveness upon inhibiting AURKA (**Figure 9L** and **Supplementary Figure 9C**). Additionally, 3D spheroid cultures were established to investigate the function of AURKA in the growth of EAC spheroids. OE33 and OE19 spheroids were treated with a vehicle control (0.1% DMSO), Aur-I at 1 µM, or 5 µM. The results demonstrated that inhibiting AURKA with Aur-I significantly (p < 0.05) reduced the spheroid area after 10 days (**Figure 9M**).

## 4 Discussion

AURKA has recently garnered attention for its atypical expression in various human tumors (3–7), suggesting its potential as a vital biomarker for diagnosing and predicting the prognosis of diverse cancers. Additionally, Lu H et al. conducted an investigation into AURKA’s function in EAC (19). Their analysis of AURKA expression and binding immunoglobulin protein (BIP) in EAC revealed a positive correlation between AURKA and a cellular phenomenon known as the unfolded protein response (UPR). This study demonstrated that AURKA played a role in regulating cell growth and development by modulating the URP pathway, as evidenced by the use of AURKA-siRNA and alisertib in EAC cells. As a result, we not only confirmed AURKA’s involvement in cellular growth in EAC but also identified other cellular pathways through comprehensive assessments, including investigations into cellular viability, colony formation, cell cycle analysis, and migration assays involving AURKA knockdown and inhibition using Aurka-Inhibitor-1.

In our current study, we scrutinized AURKA expression levels across 27 types of human cancer, revealing up-regulation in 26 cancer types and down-regulation in just one (LAML). Importantly, we uncovered that higher AURKA expression was associated with younger patients (age ≤ 65 years) and those in the M1 phase among patients with EAC, a previously unexplored aspect. Furthermore, our bioinformatic analysis unveiled AURKA’s involvement in cell cycle regulation, which was corroborated by cellular experiments showing that inhibiting AURKA expression induced cell cycle arrest in EAC cells. Lastly, we ventured into the exploration of AURKA’s function in EAC spheroids, a novel endeavor in the context of EAC. Our findings demonstrated that inhibiting AURKA significantly reduced the spheroid area in EAC cells. In summary, the novelty of our study is reflected in several aspects: 1. A comprehensive analysis of AURKA’s oncogenic role and prognostic relevance across various cancer types, highlighting its overexpression and clinical significance in diverse malignancies. 2. AURKA’s close association with immune cell infiltration, promoting tumor progression. 3. Conducting experiments in EAC, prostate cancer, and pancreatic cancer cells, revealing that inhibiting AURKA expression substantially reduced cell viability. This further confirms that the oncogenic effect of *AURKA*, reinforcing the widespread oncogenic influence of AURKA and its potential as a therapeutic target in future cancer treatment.

Most investigations have consistently demonstrated high AURKA expression in the majority of cancers, affecting tumor proliferation and invasion capabilities. Prognostic value analysis revealed that increased AURKA expression correlated with poorer outcomes in most cancers, aligning with previous studies (31–33). However, in specific cancers such as THYM, READ, COAD, OV, STAD, and LAML, our results indicated that higher AURKA expression was associated with improved prognosis. These findings were validated in GEO cohorts, although the underlying mechanisms remain undisclosed. This variability could stem from differences in the functions of AURKA and its downstream targets in various tumor types, akin to other genes such as DKK1 and Fam20C, which exhibit varying prognostic implications in different cancer types within pan-cancer studies (34, 35).

High expression of *AURKA* is reported to be significantly associated with better survival in colon cancer (36). However, the study by Hong et al. showed that AURKA induced migration and invasion in colon cancer cells (37). Similarly, the study by Chuang et al. indicated that overexpression of *AURKA* contributes to the progression of colorectal carcinoma (38). The study by Marta et al. indicated that high expression of *AURKA* was associated with better OS and progression-free survival in OV (39). On the contrary, the study by He et al. showed that *AURKA* was associated with poor prognosis in patients with OV (33). Zhou et al. (40) and Qi et al. (41) showed that *AURKA* was associated with poor prognosis of STAD and LAML, respectively. These studies reported a contradictory association between AURKA expression and these tumors.

In our current investigation, we found that AURKA exhibited significantly elevated expression levels and was correlated with OS and DSS across multiple cancer types, including esophageal carcinoma (ESCA). Our analyses further revealed that higher AURKA expression was notably present in younger patients (age ≤ 65) and those in the M1 phase among patients with EAC, providing evidence of AURKA’s potential role in promoting tumor progression. Building upon previous studies highlighting AURKA’s influence on cell proliferation and migration (42, 43), our experiments confirmed that inhibiting AURKA led to reduced cell viability and diminished cell invasion abilities in EAC, PRAD, and PAAD cells. These findings underscore the wide-ranging carcinogenic effects of AURKA.

AURKA fulfills a crucial function in cell cycle regulation, particularly in controlling cell division and spindle formation (44). GSEA indicated AURKA’s involvement in cell cycle and G2/M checkpoint pathways, which was validated through cell cycle analysis, showing that inhibiting AURKA expression induced G2/M phase arrest. Additionally, AURKA expression exhibited associations with bile acid metabolism, a factor often considered pro-carcinogenic in EAC due to its potential to induce DNA damage and influence the tumor microenvironment (45, 46). Our study unveiled AURKA’s relationships with immune and ESTIMATE scores, as well as various immune cells and immune-related regulatory genes, emphasizing its close ties to immune cell infiltration within the tumor microenvironment. Recent evidence suggests that AURKA inhibition can modulate the tumor microenvironment, enhance T-cell cytotoxicity *in vitro*, and boost anti-tumor immunity *in vivo* (16, 21).

TMB and MSI are known drivers of tumorigenesis and progression, serving as critical biomarkers for immunotherapy. Higher TMB and MSI scores are typically associated with more favorable immunotherapy outcomes (47, 48), while the TIDE score inversely correlates with clinical responses to ICI therapy (28). These indicators reveal which patients are more apt to benefit from immunotherapy. Our results demonstrated significant associations between AURKA expression and TMB, MSI, and TIDE across a broad spectrum of cancers, suggesting that AURKA expression could potentially affect the effectiveness of immunotherapy.

Furthermore, a well-established inverse correlation exists between a patient’s age and tumor metastasis (49, 50), with younger patients generally facing a higher risk of metastasis across most malignancies (49). Our study detected elevated AURKA expression in younger patients (age ≤ 65 years) compared to older patients (age > 65 years), and linked higher AURKA expression with the M1 phase in EAC. Additionally, our Boyden chamber assay affirmed that inhibiting AURKA expression reduced EAC cell invasiveness. Finally, 3D spheroid cultures provided evidence that Aur-I (AURKA inhibitor) suppressed the growth of EAC spheroids, implying that high AURKA expression might be associated with an increased risk of tumor metastasis in EAC.

## 5 Conclusions

In summary, our investigation comprehensively analyzed AURKA’s oncogenic role and prognostic significance across diverse cancer types, shedding light on its overexpression and potential clinical relevance, especially in EAC. Through bioinformatics analysis and *in vitro* experiments, we have provided compelling evidence of AURKA’s pivotal role in carcinogenesis across EAC, PRAD, and PAAD, thereby offering novel insights into future biomarkers and therapeutic strategies. Further mechanistic experiments will be undertaken to elucidate the AURKA pathways governing the progression of EAC and other cancers.

## Data availability statement 

The datasets generated in the current study are available from the corresponding author upon reasonable request.

## Ethics statement

Ethical approval was not required for the study involving humans in accordance with the local legislation and institutional requirements. Written informed consent to participate in this study was not required from the participants or the participants’ legal guardians/next of kin in accordance with the national legislation and the institutional requirements.

## Author contributions

CY performed data analysis and assisted in writing the manuscript. RT designed the study. IG and PP assisted in writing and editing the manuscript. All authors contributed to the article and approved the submitted version.

## Supplementary material

Supplementary Figure 1 Correlation of AURKA expression with overall survival **(A)** and disease-specific survival **(B)** in multiple cancer types.

Supplementary Figure 2 Relationship between AURKA expression and the ESTIMATE score.

Supplementary Figure 3 Relationship between AURKA expression and the immune score.

Supplementary Figure 4 Association between AURKA expression and immune cell infiltration.

Supplementary Figure 5 External validation of AURKA expression in the GEO cohorts. **(A-K)** LUAD, MESO, OV, PAAD, PRAD, READ, SARC, SKCM, STAD, THCA, and UCEC.

Supplementary Figure 6 External validation of AURKA prognosis in the GEO cohorts. **(A-I)** LUAD, MESO, OV, PAAD, PRAD, READ, SARC, SKCM, and STAD.

Supplementary Figure 7 Association between AURKA expression and age **(A)** and M phase **(B)** in EAC.

Supplementary Figure 8 Validation of the function of AURKA in PRAD and PAAD Cells. **(A)** AURKA mRNA level in DU145, n=5 biological replicates. **(B)** Cell proliferation of DU145 after treatment with AURKA-GapmeR transfection, n=5 biological replicates. **(C)** Cell proliferation of DU145 after treatment with Aur-I, n=3 biological replicates. **(D)** AURKA mRNA level in PaTu8988t, n=5 biological replicates. **(E)** Cell proliferation of PaTu8988t after treatment with AURKA-GapmeR transfection, n=5 biological replicates. **(F)** Cell proliferation of PaTu8988t after treatment with Aur-I, n=3 biological replicates. Data are shown as mean ± standard error of the mean. *p < 0.05, **p < 0.01; ***p <0.001; ****p < 0.0001. Aur-I: Aurora-A Inhibitor I.

Supplementary Figure 9 Images of the colony formation assay **(A)**, cell cycle assay **(B)**, and Boyden chamber assay **(C)** after cells were treated with AURKA-GapmeR or Aurora-A Inhibitor I.
