# Supplementary figures and images for: Pan-cancer analysis and in vitro validation of the oncogenic and prognostic roles of AURKA in human cancers
Source: Front Oncol. 2023 Oct 27;13:1186101. doi: 10.3389/fonc.2023.1186101 (PMC10642189; doi:10.3389/fonc.2023.1186101)

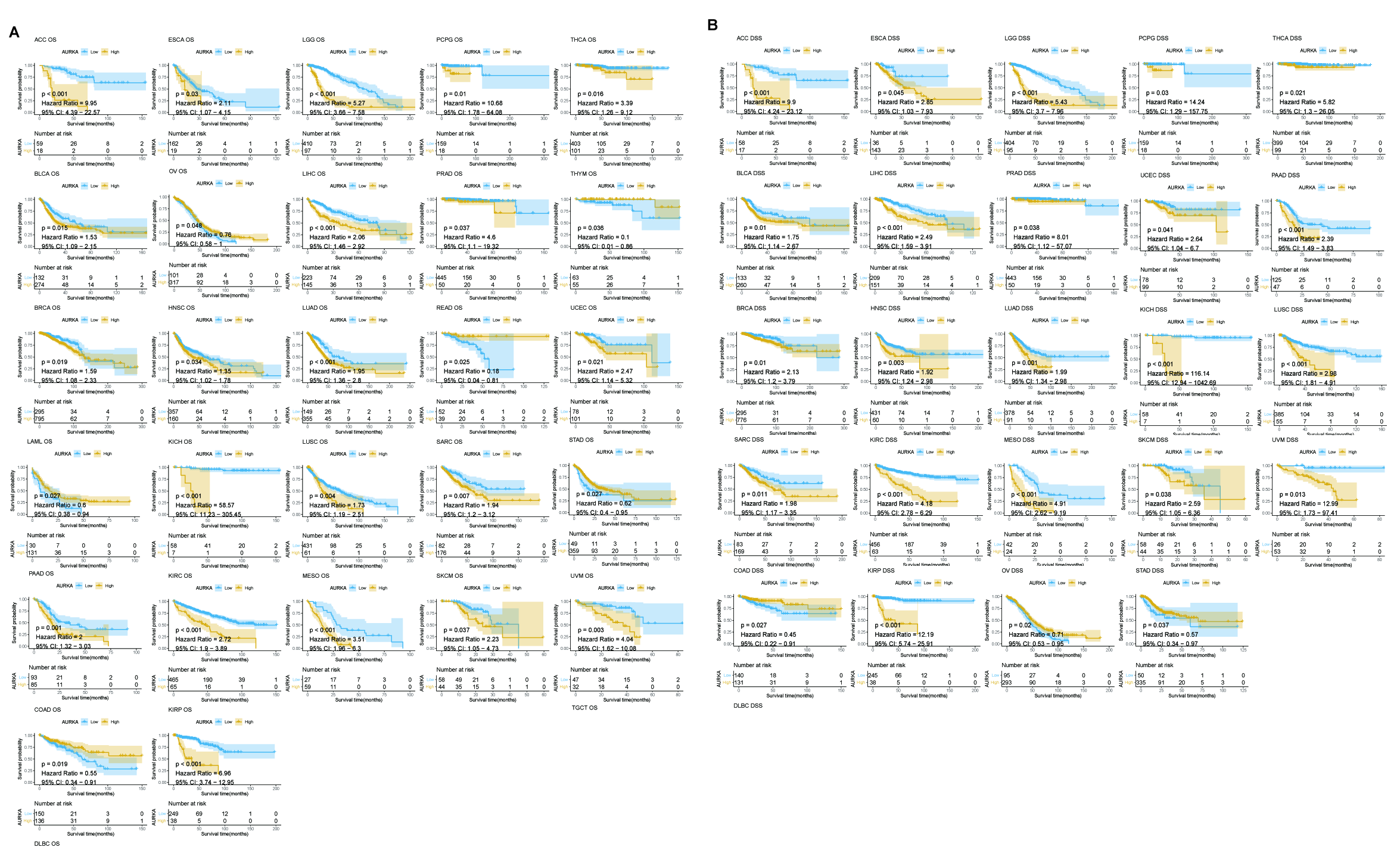

Supplement: Supplementary Figure 1 — Correlation of AURKA expression with overall survival (A) and disease-specific survival (B) in multiple cancer types. [file Image_1.tif]

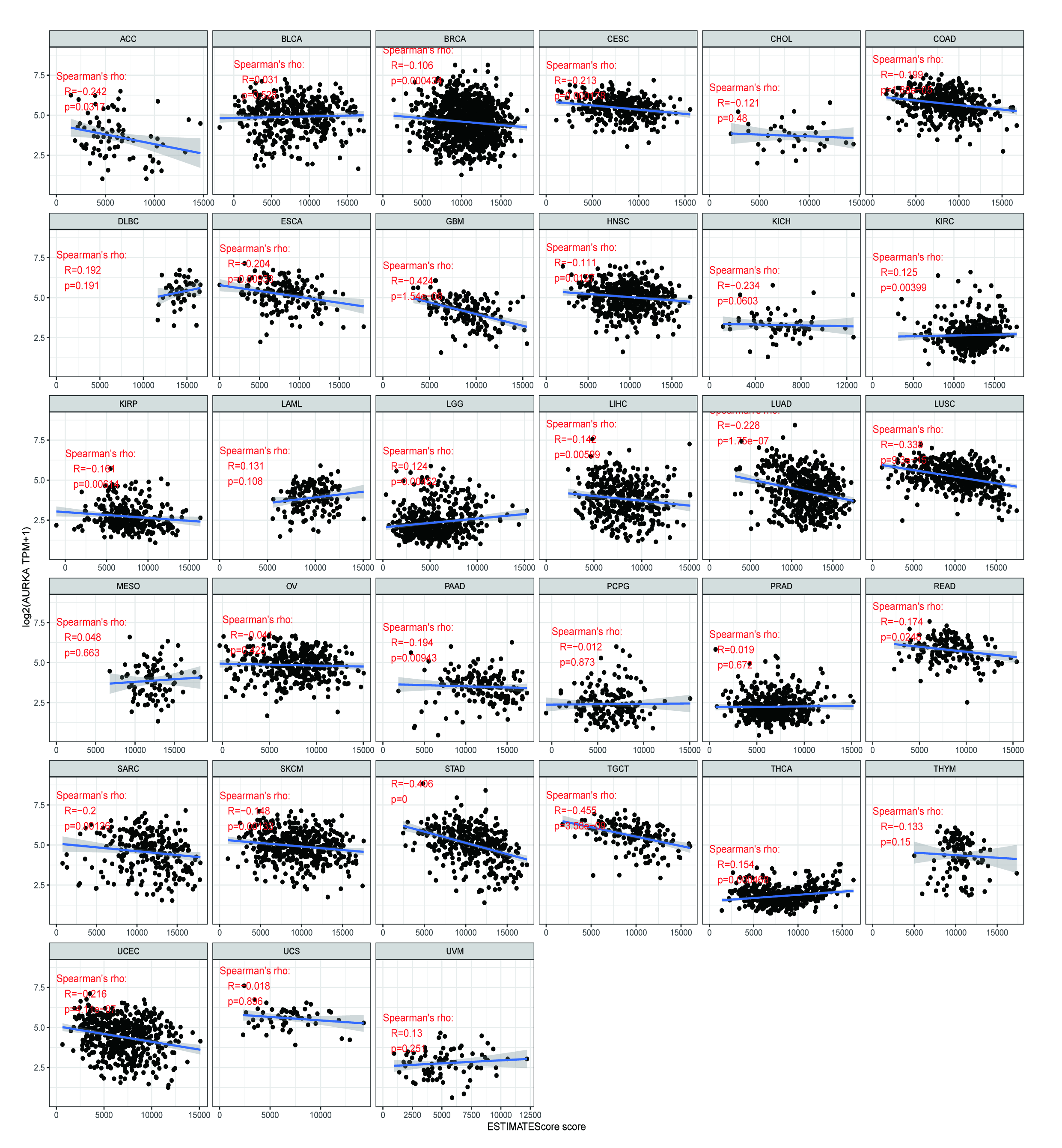

Supplement: Supplementary Figure 2 — Relationship between AURKA expression and the ESTIMATE score. [file Image_2.tif]

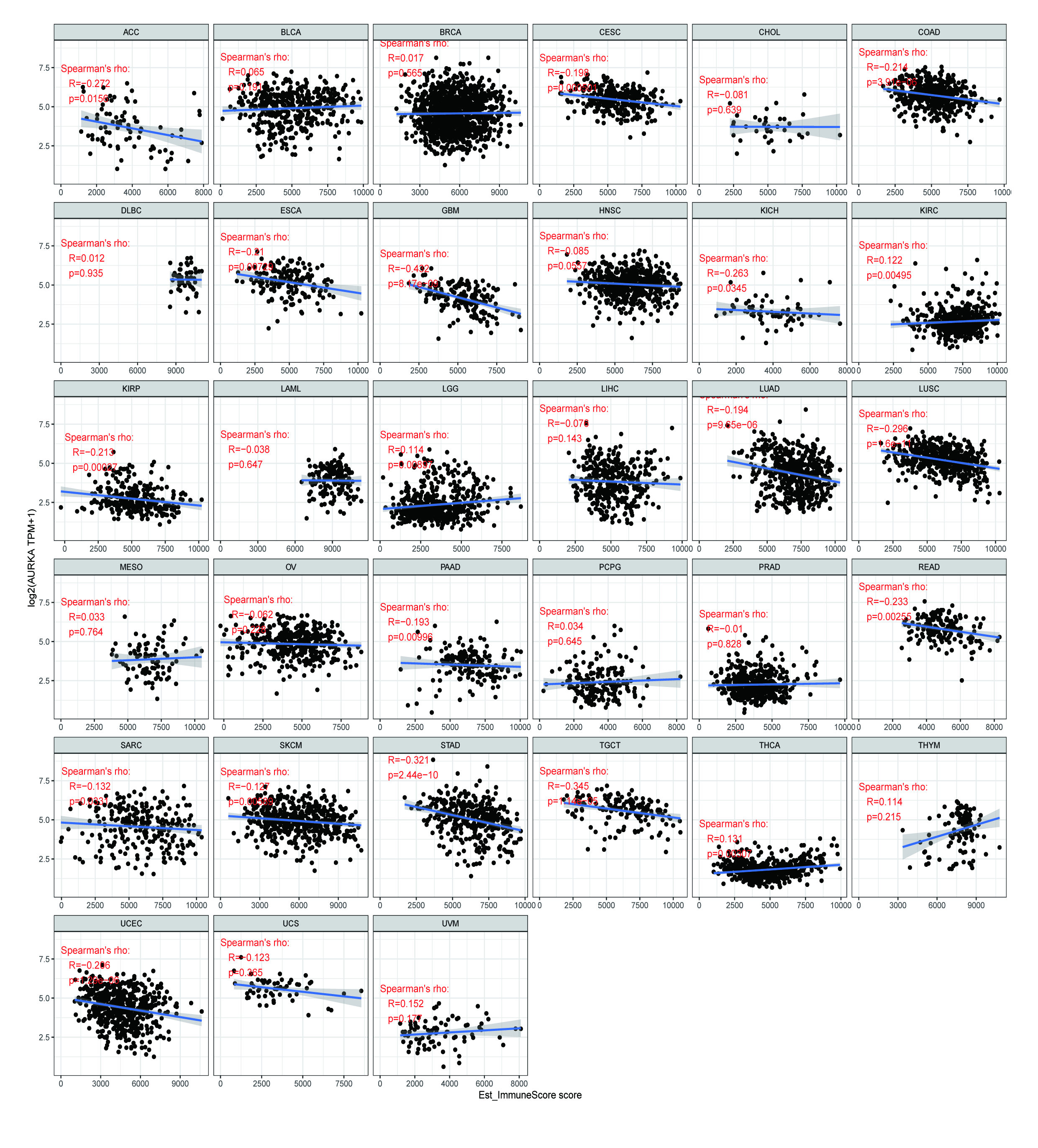

Supplement: Supplementary Figure 3 — Relationship between AURKA expression and the immune score. [file Image_3.tif]

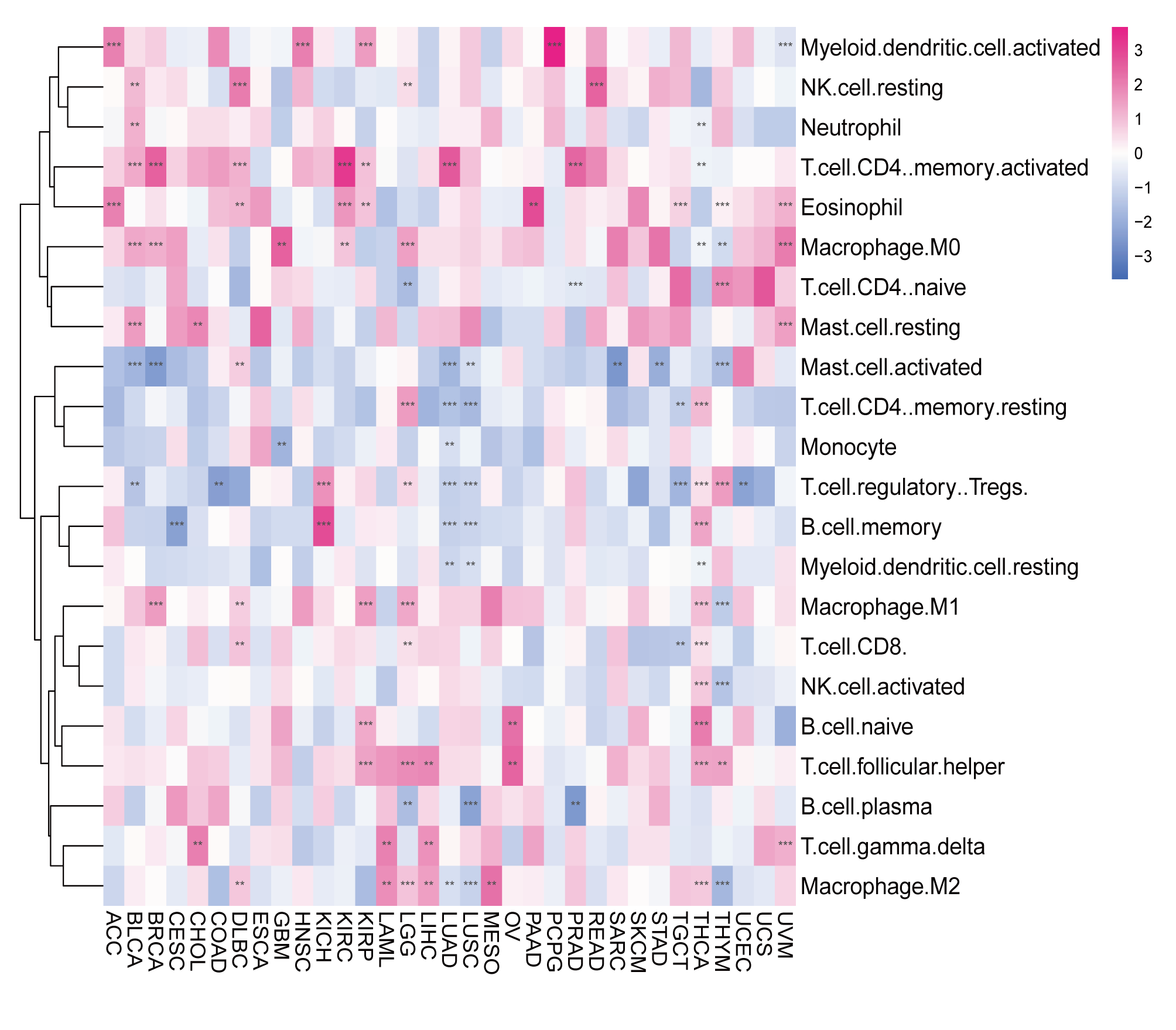

Supplement: Supplementary Figure 4 — Association between AURKA expression and immune cell infiltration. [file Image_4.tif]

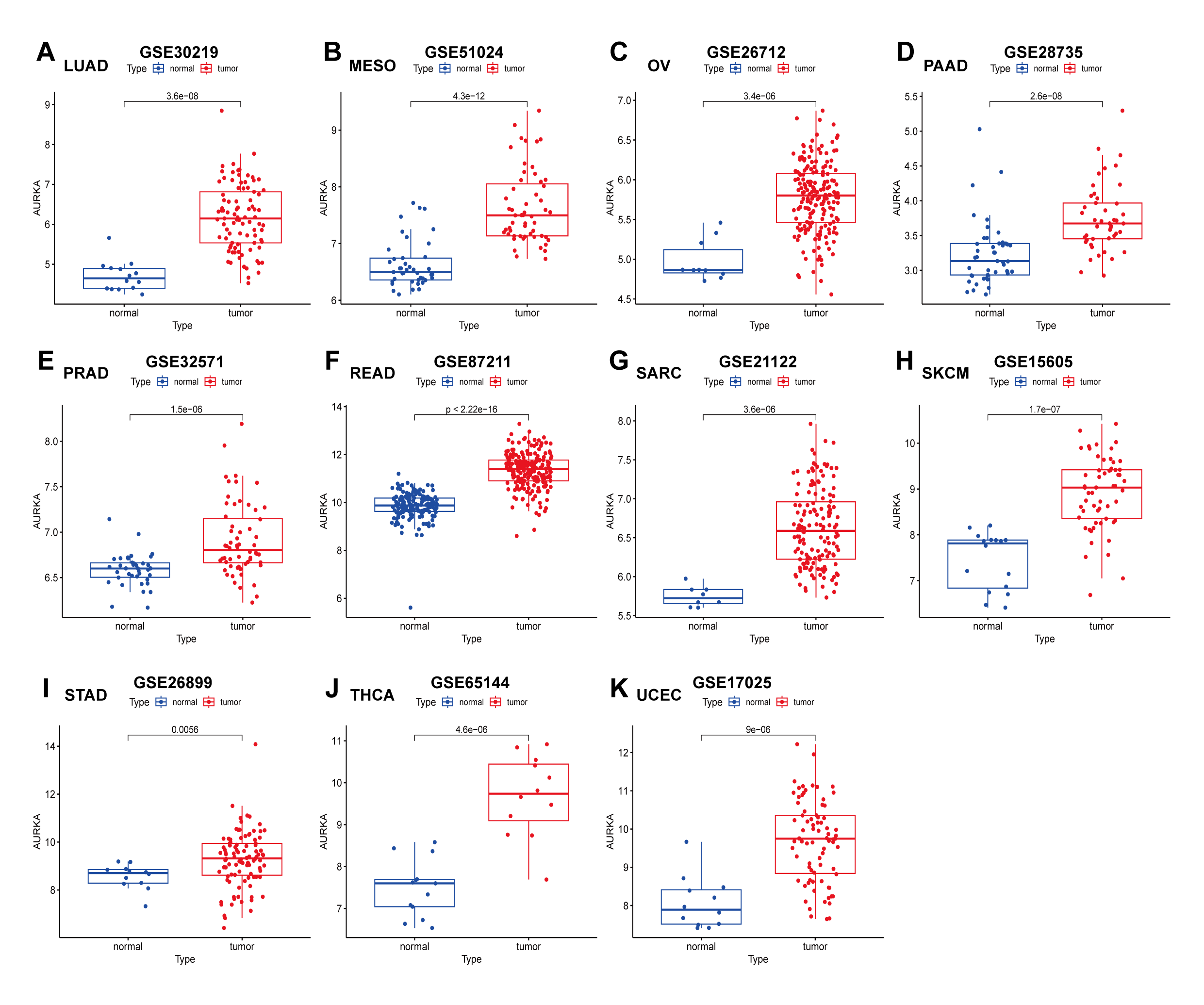

Supplement: Supplementary Figure 5 — External validation of AURKA expression in the GEO cohorts. (A-K) LUAD, MESO, OV, PAAD, PRAD, READ, SARC, SKCM, STAD, THCA, and UCEC. [file Image_5.tif]

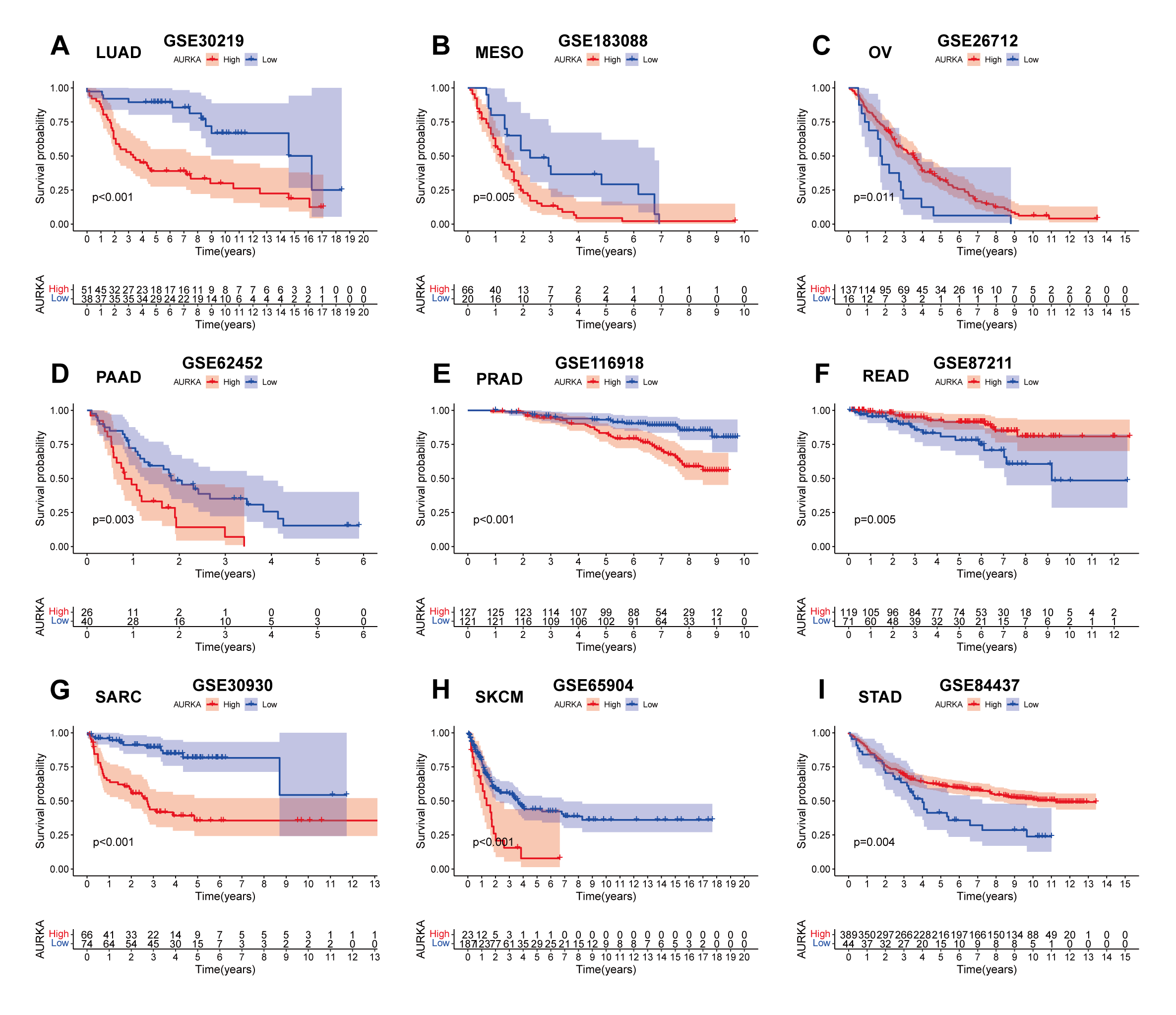

Supplement: Supplementary Figure 6 — External validation of AURKA prognosis in the GEO cohorts. (A-I) LUAD, MESO, OV, PAAD, PRAD, READ, SARC, SKCM, and STAD. [file Image_6.tif]

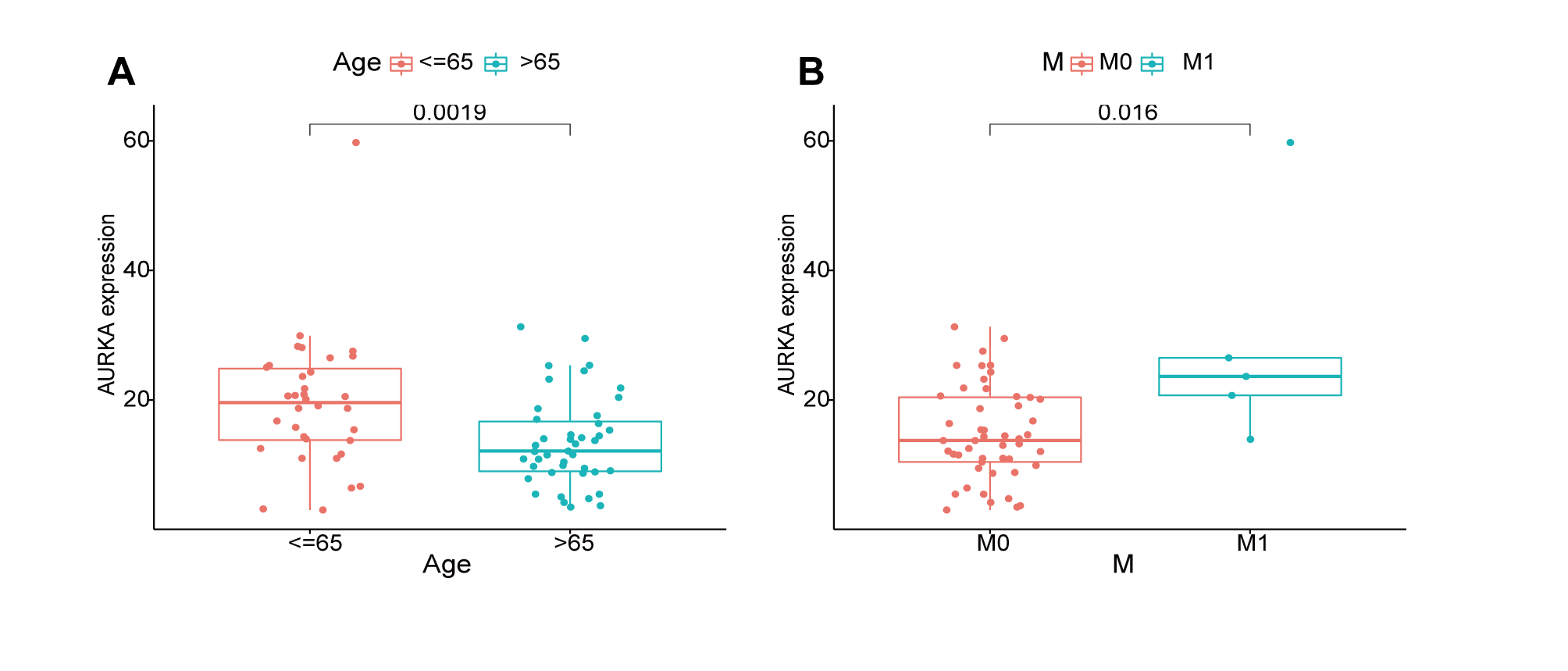

Supplement: Supplementary Figure 7 — Association between AURKA expression and age (A) and M phase (B) in EAC. [file Image_7.tif]

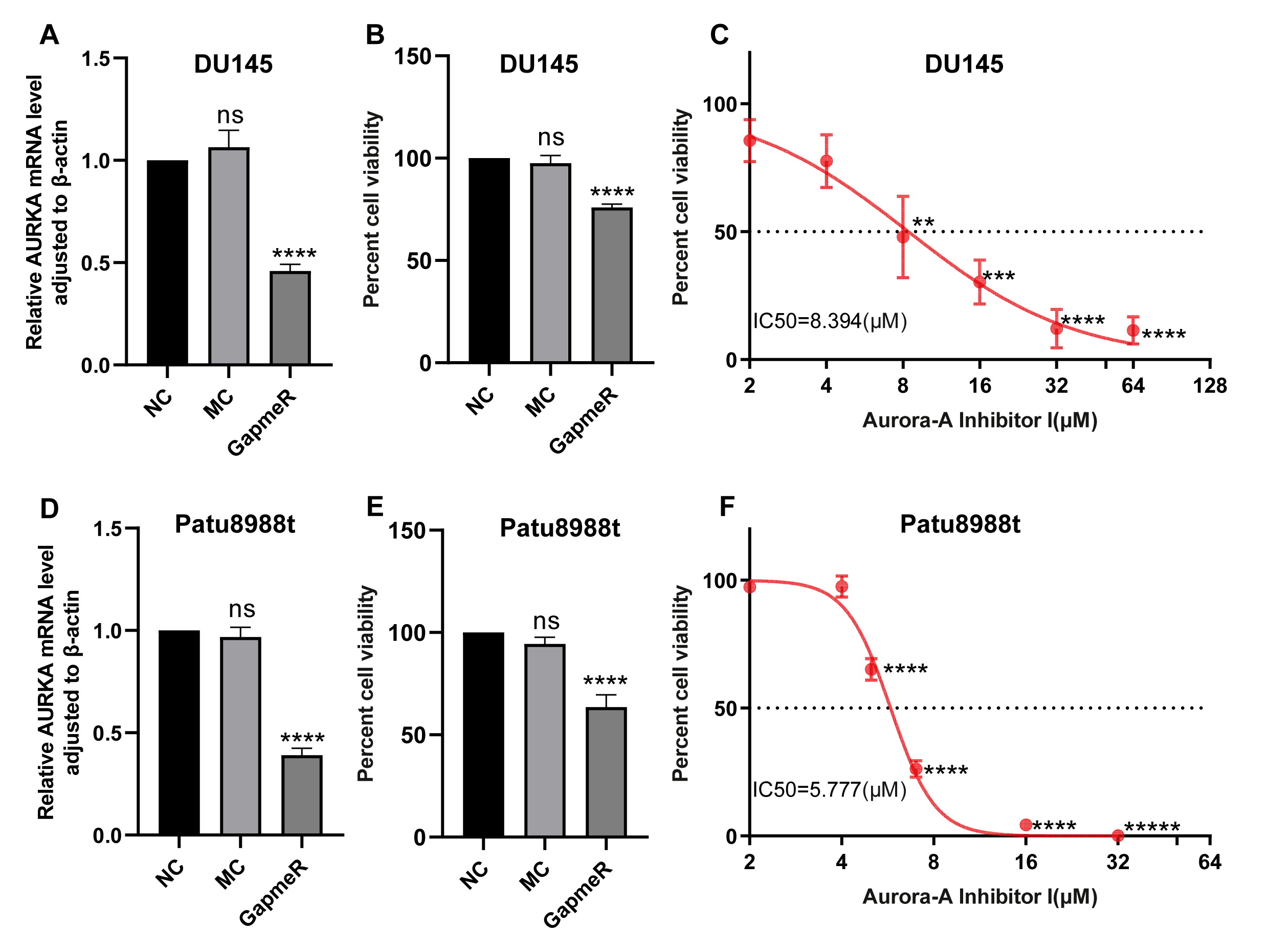

Supplement: Supplementary Figure 8 — Validation of the function of AURKA in PRAD and PAAD Cells. (A) AURKA mRNA level in DU145, n=5 biological replicates. (B) Cell proliferation of DU145 after treatment with AURKA-GapmeR transfection, n=5 biological replicates. (C) Cell proliferation of DU145 after treatment with Aur-I, n=3 biological replicates. (D) AURKA mRNA level in PaTu8988t, n=5 biological replicates. (E) Cell proliferation of PaTu8988t after treatment with AURKA-GapmeR transfection, n=5 biological replicates. (F) Cell proliferation of PaTu8988t after treatment with Aur-I, n=3 biological replicates. Data are shown as mean ± standard error of the mean. *p < 0.05, **p < 0.01; ***p <0.001; ****p < 0.0001. Aur-I: Aurora-A Inhibitor I. [file Image_8.tif]

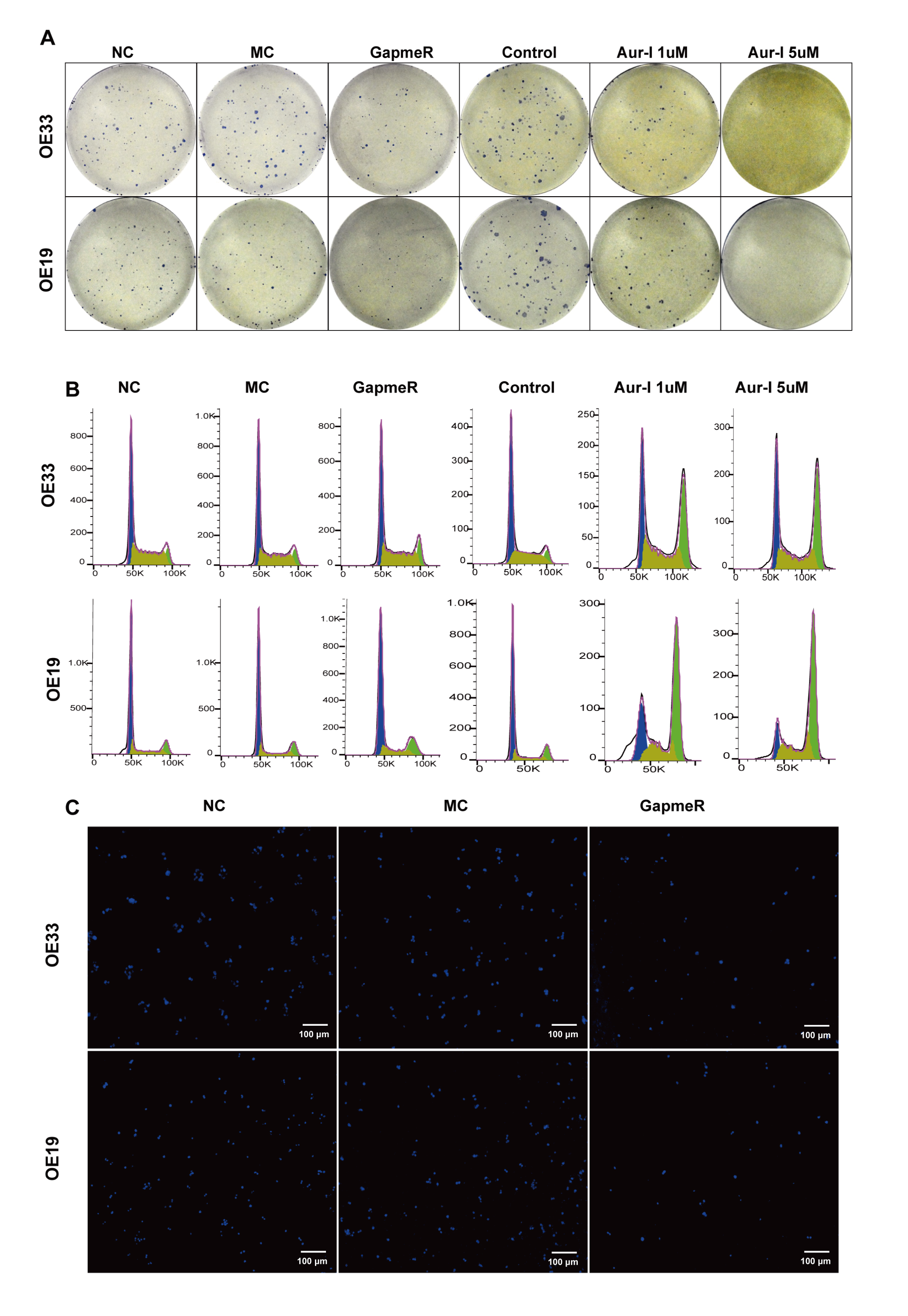

Supplement: Supplementary Figure 9 — Images of the colony formation assay (A), cell cycle assay (B), and Boyden chamber assay (C) after cells were treated with AURKA-GapmeR or Aurora-A Inhibitor I. [file Image_9.tif]
